# A Review of the Integration of Artificial Intelligence in Cardiac Electrophysiology

**DOI:** 10.3390/jcm15176754

**Published:** 2026-08-31

**Authors:** Deitrich Gerlt, Rahul Chaudhary, Oladipupo Olafiranye

**Affiliations:** 1 Department of Medicine, Methodist Dallas Medical Center, Dallas, TX 75202, USA; deitrich.gerlt@gmail.com; 2 AI-HEART Lab, Wexford, PA 15240, USA; 3 Division of Cardiology, Department of Medicine, VA Pittsburgh Medical Center, Pittsburgh, PA 15240, USA; 4 Department of Computer Science, Georgia Institute of Technology, Atlanta, GA 30332, USA; 5 Division of Cardiology, Department of Medicine, Dallas VA Medical Center, Dallas, TX 75216, USA; 6 Division of Cardiology, Department of Medicine, UT Southwestern Medical Center, Dallas, TX 75390, USA

**Keywords:** artificial intelligence, machine learning, deep learning, cardiac electrophysiology, arrhythmia, atrial fibrillation, catheter ablation, cardiac implantable electronic devices, cardiac resynchronization therapy, sudden cardiac death

## Abstract

Cardiac electrophysiology (EP) is inherently data-centric, spanning brief 12-lead electrocardiograms (ECGs), high-density electroanatomic maps, and continuous device-based monitoring. This data volume can strain provider workflows while creating an opportunity for artificial intelligence (AI). Machine learning (ML) and its deep learning subfield extract clinically actionable patterns from complex electrical signals. This narrative review summarizes contemporary AI applications across the major domains of EP. In arrhythmia detection, deep neural networks classify rhythms at a level comparable to cardiologists on internal test sets, identify occult atrial fibrillation (AF) from a normal sinus-rhythm ECG, and, through consumer wearables, extend screening to ambulatory populations. In catheter ablation, an AI algorithm that adjudicates intracardiac electrogram dispersion improved single-procedure freedom from AF in a randomized trial of persistent AF, and ML models help predict arrhythmia recurrence; we distinguish these from adjacent non-AI technologies, such as computed-tomography integration and three-dimensional mapping, that reduce fluoroscopy but are not themselves AI. In cardiac implantable electronic devices (CIEDs), AI-based filtering lowers false-positive alert burden, and multi-parametric algorithms provide earlier prediction of heart-failure decompensation. ML models may refine patient selection for cardiac resynchronization therapy (CRT) and, using late-gadolinium-enhancement cardiac magnetic resonance, may sharpen arrhythmic-risk and implantable cardioverter-defibrillator (ICD) decision-making. AI-enhanced ECG broadens the standard ECG into a low-cost screening tool for channelopathies, dyskalemias, and ventricular dysfunction. Important barriers remain, including limited external validation, incomplete explainability, and a scarcity of prospective outcome trials.

## 1. Introduction

Cardiac electrophysiology (EP) is an inherently data-centric discipline in which arrhythmias are interpreted from electrical signals with non-linear dynamics on the electrocardiogram (ECG). The modern EP landscape is characterized by an abundance of data, ranging from high-density electroanatomic mapping systems that generate thousands of points per minute to implantable loop recorders (ILRs), which provide continuous long-term rhythm monitoring. While this surplus of data can present a logistical challenge for clinicians, it simultaneously offers an opportunity for advanced, data-driven diagnostics [1,2].

Artificial intelligence (AI), encompassing traditional machine learning (ML) and modern deep learning (DL), provides a computational framework for analyzing this information. The purpose of this review is to examine the applications of AI across the principal domains of EP: arrhythmia detection and screening, catheter ablation, cardiac implantable electronic devices (CIEDs), resynchronization therapy, arrhythmic risk stratification, and genetic and metabolic screening. Across cardiovascular medicine, more broadly, reported AI models span a wide range of diagnostic performance (reported sensitivities of roughly 0.28–0.99 and specificities of 0.68–0.93), reflecting both the promise and the heterogeneity of the field [3].

## 2. Review Approach

This is a narrative review intended to synthesize representative, high-impact work rather than to provide a systematic quantitative synthesis. We searched PubMed/MEDLINE for English-language articles published up to 2025, combining terms for artificial intelligence, machine learning, and deep learning with terms for the EP domains above (for example, “electrocardiography,” “atrial fibrillation,” “catheter ablation,” “implantable loop recorder,” “cardiac resynchronization therapy,” “implantable cardioverter-defibrillator,” “sudden cardiac death,” “hyperkalemia,” and “long QT syndrome”). Reference lists of retrieved articles and recent society statements were screened for additional sources. Studies were selected by author consensus to illustrate the range of clinical applications, prioritizing landmark development and validation studies, randomized trials where available, systematic reviews, and contemporary society guidance. Because the goal was the breadth of clinical illustration across heterogeneous domains and data types, a narrative rather than systematic format was chosen; the attendant limitations, including the potential for selection bias, are discussed in Section 11. Table 1 summarizes the principal studies discussed below, including their clinical domain, input data type, model class, cohort size, validation approach, main performance metrics, external-validation status, and key limitations.

## 3. Machine Learning Fundamentals

Machine learning was described in 1959 by Arthur Samuel, who proposed that programming computers to learn from experience should reduce the need for detailed task-specific programming [21]. In contemporary practice, ML systems fall into three broad paradigms that differ in the training signal they use [22,23].

**Figure 1:**
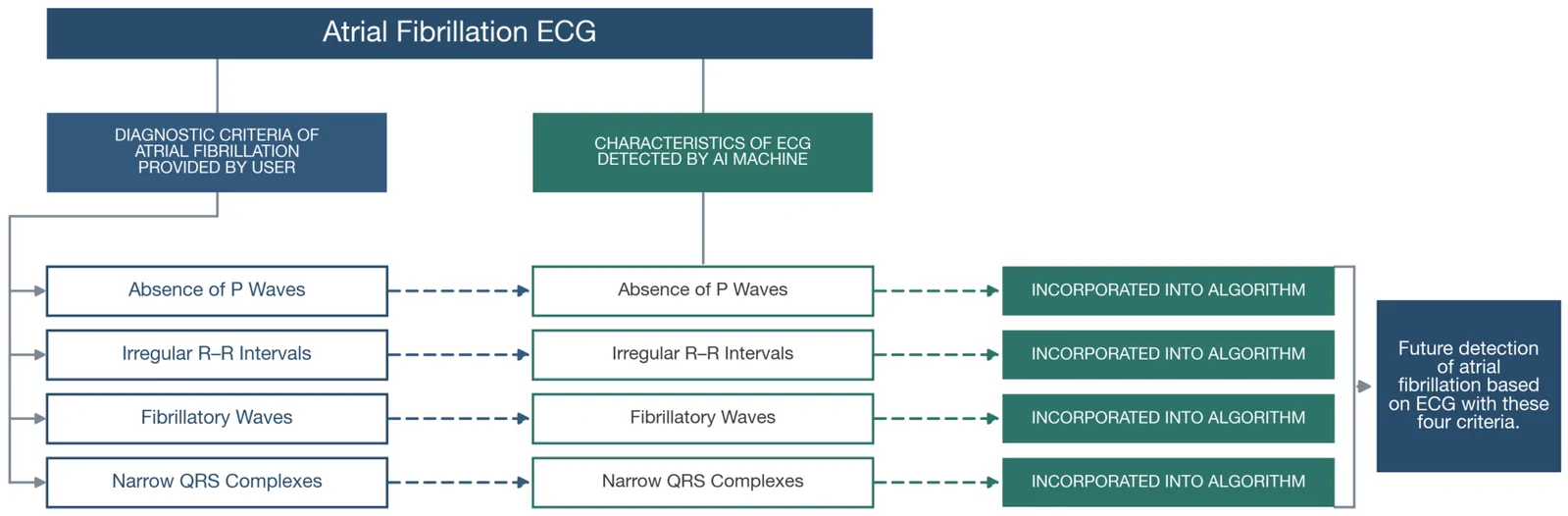
Supervised machine learning applied to an atrial fibrillation ECG. The diagnostic criteria of atrial fibrillation-absence of P waves, irregular R–R intervals, fibrillatory waves, and narrow QRS complexes are provided by the user (the labeled ground truth) and incorporated into the algorithm, which then detects atrial fibrillation on subsequent tracings. If the labeled examples are confounded (for example, if most share a common heart rate), the model may instead learn a spurious cue—a failure mode termed shortcut learning.

In supervised learning, the model is trained on labeled examples (for example, ECGs each paired with a confirmed, human-adjudicated diagnosis) and learns a mapping from input to output by iteratively adjusting internal weights to reduce error, a process known as gradient descent with backpropagation. A representative challenge is teaching a model to recognize atrial fibrillation (AF) from its true diagnostic criteria (absent P waves, fibrillatory waves, irregular R-R intervals, and narrow-complex QRS complexes) rather than from a confounded cue. If many AF examples in the training set happen to share a heart rate near 88 beats per minute, the model may treat that rate as a defining feature; this failure mode is termed shortcut learning, and it would cause the model to miss AF with a rapid or slow ventricular response (Figure 1). Deep learning, a subfield of ML that uses multi-layer neural networks, differs in that discriminative features are learned directly from the raw signal rather than being hand-specified; a deep network may correctly weight irregular R–R intervals or, if the data are confounded, may latch onto the spurious 88-beats-per-minute cue.

**Figure 2:**
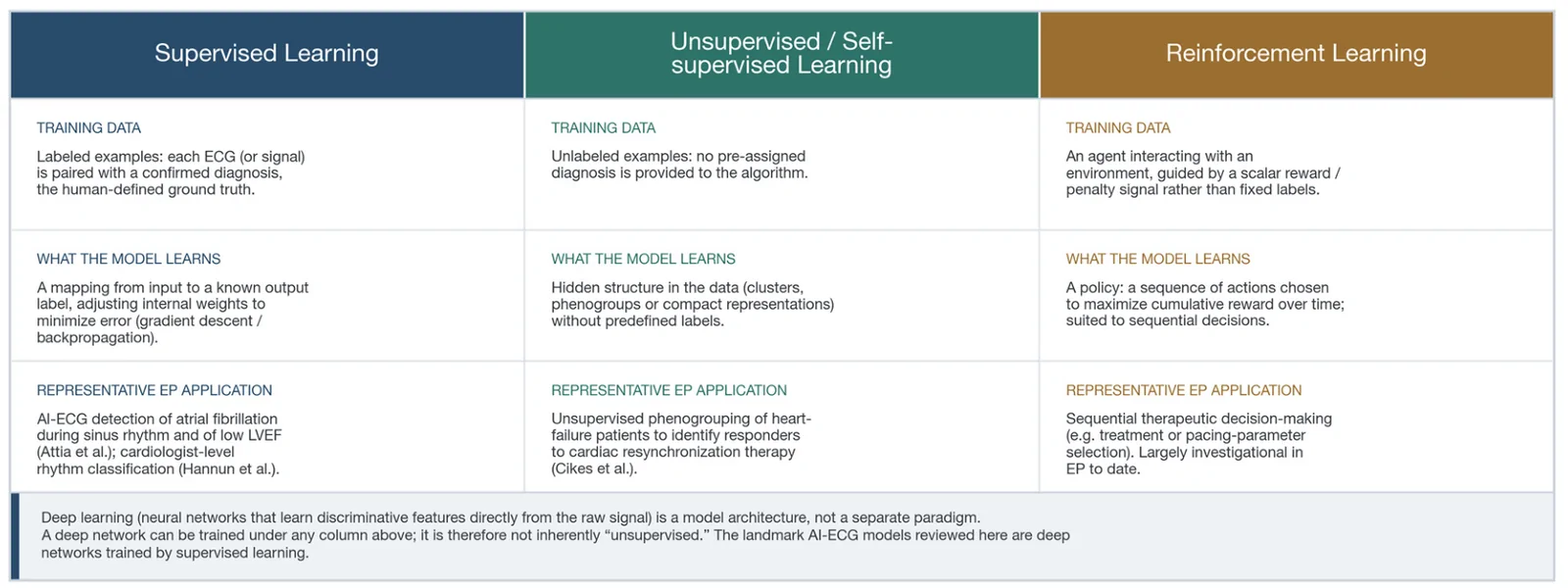
Machine-learning paradigms relevant to cardiac electrophysiology. Supervised learning maps labeled inputs to known outputs; unsupervised and self-supervised learning discover latent structure in unlabeled data; reinforcement learning optimizes a sequential action policy from a reward signal. Deep learning—neural networks that learn features directly from the raw signal—is a model architecture that can be trained under any of these paradigms and is not itself a distinct or inherently “unsupervised” category. Representative electrophysiology applications are shown for each paradigm [4,5,16].

In unsupervised (and the closely related self-supervised) learning, the model is given unlabeled data and discovers latent structure (clusters, phenogroups, or compact representations) without predefined labels. In reinforcement learning, an agent interacts with an environment and learns a policy that maximizes a cumulative reward signal, a paradigm suited to sequential decisions such as therapy selection (Figure 2).

Two points warrant emphasis because they are frequently conflated. First, deep learning is a model architecture, not a training paradigm: a deep neural network can be trained under supervised, unsupervised, self-supervised, or reinforcement objectives, and is therefore not inherently “unsupervised.” The landmark AI-ECG models discussed in this review, including those of Attia and Hannun, are deep networks trained by supervised learning [4,5]. Second, the choice of paradigm is dictated by the available labels and the clinical question, not by the depth of the network.

## 4. Arrhythmia Detection and Screening

AI applied to arrhythmia detection has evolved from simple threshold-based algorithms to deep neural networks that classify rhythms at a level comparable to cardiologists on internal test data. In a landmark study, a deep neural network trained on 91,232 single-lead ambulatory ECG recordings classified 12 rhythm classes with performance approaching that of board-certified cardiologists [4]. On the internal test set, the network achieved an area under the receiver-operating-characteristic curve of 0.97 and an F1 score of 0.837, exceeding the average individual cardiologist’s F1 score of 0.780 against a consensus-committee reference. These figures are point estimates reported without 95% confidence intervals and reflect performance on an internally annotated test set; independent prospective validation across diverse populations remains limited, so the results are best read as matching—and in specific tasks, approaching or exceeding—cardiologist-level performance under study conditions rather than as established superiority in routine practice. Such automated triage is particularly relevant for continuous monitors (for example, ILRs and Holter monitors), where data volume contributes to clinician fatigue and incomplete review, and it can prioritize critical arrhythmias such as ventricular tachycardia and high-grade atrioventricular (AV) block for immediate human attention while filtering noise.

Beyond rhythm classification, AI can detect occult or “invisible” AF from a standard sinus-rhythm ECG. Using 649,931 ECGs from 180,922 patients, a convolutional neural network (CNN) developed at the Mayo Clinic identified paroxysmal AF from 10 s, 12-lead recordings acquired during normal sinus rhythm [5]. On a single ECG, the model achieved an area under the curve (AUC) of 0.87 (95% CI 0.86–0.88), sensitivity of 79.0% (95% CI 77.5–80.4), and specificity of 79.5% (95% CI 79.0–79.9); across all ECGs from a one-month window, the AUC rose to 0.90 (95% CI 0.90–0.91). These figures derive from a single-center internal test set; external and prospective screening cohorts have reported lower discrimination (AUC near 0.62 in one population study), a reminder that internal accuracy may not carry over to general screening populations. Nonetheless, the approach reframes the ECG from a snapshot of current electrical activity into a tool for identifying patients at elevated future risk.

Consumer wearables represent a third frontier. The Apple Heart Study used a smartwatch optical (photoplethysmographic) sensor and a rule-based algorithm to screen 419,297 participants for pulse irregularity [6]. Over a median of 117 days, 2161 participants (0.52%) received an irregular-pulse notification; of those, 450 returned an analyzable ambulatory ECG patch, and AF was confirmed in 34%. Among notified participants who returned a patch, the positive predictive value was 0.84. This value should be interpreted cautiously because only about 21% of notified participants (450 of 2161) returned an analyzable patch, so it rests on a small, self-selected subset. The study demonstrates that a wearable can screen a large, real-world population using a simple pulse signal, but it does not establish cost-effectiveness, which would require accounting for the downstream burden of evaluating false-positive notifications at scale.

## 5. Catheter Ablation

### 5.1. AI-Driven Mapping and Target Selection

Identifying optimal ablation targets is challenging, particularly in persistent AF, where pulmonary vein isolation (PVI) alone is frequently insufficient. In a proof-of-concept study, a CNN classified rotational activation sites (rotors) from panoramic bi-atrial recordings in 35 patients with 95% accuracy and operated with a decision logic that mirrored expert evaluation (C-statistic 0.96) despite never being given those rules explicitly [7].

The most direct clinical evidence to date comes from the TAILORED-AF randomized controlled trial [8]. In this multi-center, double-blind, superiority trial, 370 patients with persistent or long-standing persistent AF were randomized to a tailored ablation targeting AI-identified areas of spatio–temporal electrogram dispersion in addition to PVI (tailored arm, *n* = 187) or to PVI alone (anatomical arm, *n* = 183). The AI system (Volta AF-Xplorer, formerly VX1, version 1.3 or later; Volta Medical, Marseille, France) is an ensemble classifier, combining a gradient-boosting model and a CNN trained on more than 275,000 annotated intracardiac electrograms, that adjudicates dispersion in real time to standardize target identification across operators and centers. The trial met its primary efficacy endpoint: single-procedure freedom from AF at 12 months was 88% in the tailored arm versus 70% in the anatomical arm (hazard ratio 0.30, 95% CI 0.21–0.57; log-rank *p* < 0.0001), with the benefit being most pronounced in patients with longer AF duration. Several caveats temper enthusiasm: there was no significant difference in freedom from any atrial arrhythmia after a single procedure (60% versus 60%), recurrences in the tailored arm were more often organized atrial tachycardias, procedure and ablation times were roughly twice as long, and the ML model had to be frozen in 2020, underscoring the difficulty of deploying evolving algorithms within fixed-protocol trials. Even so, TAILORED-AF is the first large-scale randomized trial in interventional EP to show superior efficacy from an AI-guided strategy.

Beyond target selection, ML can help predict ablation outcomes. A systematic review and meta-analysis reported that ML models integrating clinical, imaging (for example, left atrial fibrosis on magnetic resonance imaging (MRI)), and electrophysiological inputs outperformed conventional recurrence scores such as APPLE, CAAP-AF, and MB-LATER, with a mean AUC of approximately 0.81 [9]. This pooled figure should be read as an average across heterogeneous models and cohorts rather than a single validated performance estimate; heterogeneity was substantial, and most included models were tested only internally, with few externally or prospectively validated.

### 5.2. Adjacent Digital Technologies (Not AI-Based)

Several technologies commonly grouped with “AI in ablation” are more accurately described as digital image-integration and mapping advances that do not themselves employ AI, and we distinguish them here to avoid conflation. The integration of pre-procedural computed-tomography (CT) anatomy into an electroanatomic mapping system has been associated with reduced fluoroscopy time and improved outcomes: in one study of 94 patients, PVI success was comparable between groups, but fluoroscopy time was lower in the CT-integration group (49 ± 27 min) than in the independent three-dimensional (3D) mapping group (62 ± 26 min) [24]. Similarly, in 295 patients, fluoroscopy-integrated 3D mapping significantly reduced radiation exposure during AF and ventricular tachycardia ablation without compromising safety [25]. These reductions in fluoroscopy are attributable to 3D mapping and image registration rather than to AI; we include them for completeness and to clarify the boundary between AI-enabled and adjacent digital tools.

## 6. Cardiac Implantable Electronic Devices (CIEDs)

### 6.1. Alert Triage

The remote monitoring of CIEDs generates large volumes of transmissions, many of them non-actionable—particularly false-positive AF detections from ILRs reflecting atrial ectopy or signal artifact. In a multi-center evaluation of an ML reclassification approach applied to ILR transmissions, 370 patients generated 3755 episodes; among the 2821 episodes analyzable by the algorithm, 1227 (43%) were reclassified as normal rhythm, substantially reducing the volume requiring clinical review [10]. In a separate validation of 1500 ILR-detected AF episodes, a deep-neural-network filter improved the positive predictive value of ILR-detected AF from 53.9% to 74.5% with negligible loss of true AF, reducing adjudication time and alarm fatigue [11].

### 6.2. Multi-Parametric Prediction of Heart-Failure Decompensation

Contemporary CIEDs also function as multi-parametric physiological sensors, and two composite algorithms illustrate how these signals are combined. The HeartLogic index (developed and validated in the MultiSENSE study) integrates device-based heart sounds (including the third heart sound), respiration, thoracic impedance, heart rate, and activity into a single composite score; when the score crosses a programmable threshold, an alert is generated. Across 900 patients followed for one year, HeartLogic detected 70% of adjudicated heart-failure events with a median warning time of 34 days while maintaining a low unexplained-alert rate (1.47 per patient-year) [12]. A conceptually similar tool, the Triage Heart Failure Risk Status (Triage-HF), combines up to nine device-derived parameters, including AF burden, ventricular rate during AF, percentage of biventricular pacing, thoracic impedance (OptiVol), patient activity, and night heart rate, using a Bayesian belief network to classify a patient’s 30-day risk of heart-failure hospitalization as low, medium, or high. In the prospective TRIAGE-HF study of 100 CRT-defibrillator or ICD recipients, a high-risk status corresponded to clinical features of worsening heart failure [26]; in a larger real-world analysis of 429 patients, a high-risk status conferred 37.3% sensitivity and 86.2% specificity for 30-day all-cause hospitalization, and a non-high status carried a 97.5% negative predictive value [13]. Together, these tools shift device-based monitoring from reactive toward anticipatory management, although their high false-positive rates and the absence of large, randomized outcome trials remain limitations.

### 6.3. Patient Selection for Cardiac Resynchronization Therapy

Guideline criteria for cardiac resynchronization therapy (CRT), centered on QRS duration and bundle-branch morphology, still leave a substantial minority of recipients without benefit, motivating ML approaches to responder identification. In a model-development analysis of the COMPANION trial, ML models trained on pre-implant characteristics predicted 12-month death or heart-failure hospitalization after CRT; in 595 CRT-defibrillator patients, the best model achieved an AUC of 0.74 and stratified patients into risk quartiles [14]. The single-center SEMMELWEIS-CRT score, developed in 1510 patients using 33 pre-implant clinical variables, predicted all-cause mortality after CRT with an internally calculated concordance statistic of approximately 0.69 and has since been externally validated [15]. A complementary, unsupervised approach illustrates responder identification directly: applying multiple-kernel learning and k-means clustering to clinical and echocardiographic (left ventricular deformation) data from 1106 MADIT-CRT participants, investigators derived mutually exclusive phenogroups, and two phenogroups enriched for known predictors of response derived substantially greater benefit from CRT-defibrillator therapy (hazard ratios 0.35 and 0.36 for the primary outcome) than the others (interaction *p* = 0.02) [16]. Across these studies, discrimination is modest to moderate, and no model is yet strong enough to guide CRT selection on its own; collectively, they indicate that pre-implant data add information beyond conventional ECG subgrouping.

## 7. Arrhythmic Risk Stratification and Sudden Cardiac Death

Selection for a primary-prevention implantable cardioverter-defibrillator (ICD) currently rests largely on left ventricular ejection fraction (LVEF), a blunt instrument that both misses arrhythmic deaths among patients with preserved LVEF and treats many patients who never receive appropriate therapy. AI applied to cardiac imaging and the ECG is being explored to sharpen this decision. In a widely cited study, the Survival Study of Cardiac Arrhythmia Risk (SSCAR) trained a deep neural network on raw late-gadolinium-enhancement (LGE) cardiac magnetic resonance images, which visualize myocardial scarring together with clinical covariates to predict patient-specific survival free of arrhythmic death in ischemic cardiomyopathy [17]. The model produced individualized 10-year survival curves with uncertainty estimates, achieving concordance indices of 0.83 on internal validation and 0.74 on an independent test set (10-year integrated Brier scores 0.12 and 0.14), and outperformed survival models built from clinical covariates alone. Consistent with this direction, a 2025 scientific statement from the European Heart Rhythm Association, the Heart Rhythm Society, and the ESC Working Group on E-Cardiology notes that AI combining ECG and clinical data can predict non-arrhythmic mortality to aid ICD selection (reported AUROC approximately 0.8) and that remote-monitoring device data can predict imminent ventricular arrhythmia [23]. These models remain largely single- or few-center and retrospective; the statement emphasizes that transparent reporting and external validation are prerequisites for clinical adoption.

## 8. Genetic and Metabolic Screening

AI-ECG has expanded to screening for silent channelopathies and metabolic derangements. For congenital long QT syndrome, a deep neural network trained on more than 1.6 million 12-lead ECGs from 538,200 patients estimated the corrected QT interval with high precision (mean difference −1.76 ms versus cardiologist over-read) and detected clinically relevant prolongation (QTc ≥ 500 ms) with an AUC of 0.97; performance was maintained on tracings from a mobile ECG device (80% sensitivity, 94% specificity) [18]. This suggests that the network detects subtle T-wave morphology beyond simple repolarization duration, offering a potential non-invasive screening tool for relatives of sudden-cardiac-death victims.

AI has likewise been applied to dyskalemias. Conventional ECG signs such as peaked T waves are insensitive and often late. The ECG12Net deep-learning model, trained on 66,321 ECG records from more than 40,000 patients, was developed to detect both hypokalemia and hyperkalemia and to estimate serum potassium non-invasively [19]. In a human–machine comparison, the model outperformed emergency physicians and cardiologists for hyperkalemia detection (AUC 0.958) and estimated serum potassium with a mean absolute error of 0.531 mEq/L, with comparable capability for hypokalemia. Such tools could reduce repetitive point-of-care potassium testing and enable closer non-invasive surveillance in patients with end-stage renal disease or on complex heart-failure regimens involving mineralocorticoid receptor antagonists.

## 9. Structural and Electrophysiological Overlap

The intersection of structural heart disease and EP is a fertile domain for AI. A prominent example is AI-ECG for screening asymptomatic left ventricular systolic dysfunction. In a Mayo Clinic development and validation study using paired 12-lead ECGs and echocardiograms, a CNN identified LVEF ≤ 35% from the ECG alone with an AUC of 0.93, sensitivity 86.3%, and specificity 85.7% in an independent test set of 52,870 patients [20]. Among patients without low LVEF on the index echocardiogram, a positive AI-ECG screen was associated with a 4.1-fold higher risk of subsequently developing LVEF ≤ 35%, suggesting that the model detects early or subclinical substrates; the ECG can thus serve as a low-cost rule-in trigger for confirmatory echocardiography.

## 10. Barriers to Clinical Implementation

The performance figures above are encouraging, but several barriers separate these models from routine use, and they recur across every domain reviewed here rather than in any single application. The first is explainability: many deep-learning models behave as “black boxes,” producing outputs without a transparent account of the driving features, which complicates clinician trust and regulatory audit. Saliency maps and Shapley additive explanation (SHAP) values offer partial insight but do not fully resolve the problem. The second is algorithmic bias and generalizability: a model learns the patterns of its training data, so underrepresented groups may be served less well, and performance can fall when a model is applied to a new population, as seen when sinus-rhythm AF models were tested in external screening cohorts (Section 4), when CRT models showed only modest discrimination (Section 6), when the dispersion-guided ablation strategy produced more organized atrial tachycardias and longer procedures (Section 5), and when potassium-estimation models must be shown to hold across laboratories and populations (Section 8). Careful subgroup reporting and external validation are therefore essential. The third is the evidence base itself: most studies report model accuracy on retrospective data, and prospective randomized trials linking these tools to hard outcomes (fewer strokes, fewer heart-failure admissions, longer survival) remain rare, with TAILORED-AF a notable exception. Strong discrimination on a test set does not guarantee clinical benefit; until outcome trials accumulate, most tools are best framed as supportive rather than definitive. Workflow integration, regulatory clearance, and clinician trust add further hurdles.

## 11. Limitations

This review has several limitations. It is a narrative review; although we describe our search sources and selection approach (Section 2), we did not register a protocol, predefine strict inclusion and exclusion criteria, or perform a quantitative synthesis, so selection bias is possible and some relevant work may not be represented. The evidence base is itself limited: many cited studies are single-center and retrospective, and reported metrics frequently derive from internal test sets rather than external or prospective validation, so the numbers may overstate real-world performance. The findings here should therefore be read as a survey of a fast-moving field rather than as practice guidance.

## 12. Conclusions

Artificial intelligence is modernizing cardiac electrophysiology by converting expanding volumes of ECG, mapping, and device-derived data into actionable insight. AI has improved rhythm interpretation, enabled the AI-guided ablation of persistent AF with superior single-procedure efficacy in a randomized trial, and enhanced ML-based recurrence prediction; adjacent digital technologies such as CT integration and 3D mapping have separately reduced procedural radiation. In CIED management, AI-based filtering has reduced false-positive alert burden while multi-parametric algorithms provide earlier warning of heart-failure decompensation. ML models may add information for CRT candidate selection and, using LGE cardiac magnetic resonance, for arrhythmic risk and ICD decision-making in primary prevention, though current discrimination is modest and not yet sufficient to guide decisions alone. AI-ECG broadens ECGs’ role as a low-cost screening tool for channelopathies, dyskalemias, and ventricular dysfunction. These advances remain tempered by real barriers: most evidence is retrospective and single-center, external validation is limited, explainability is incomplete, and prospective trials linking these tools to improved outcomes are still needed.

## Figures and Tables

**Table 1:** Summary of principal studies of artificial intelligence in cardiac electrophysiology discussed in this review. AF, atrial fibrillation; AUC, area under the receiver-operating-characteristic curve; CI, confidence interval; CIED, cardiac implantable electronic device; CM, cardiomyopathy; CNN, convolutional neural network; CRT, cardiac resynchronization therapy; DL, deep learning; F1, harmonic mean of precision and recall; HF, heart failure; HR, hazard ratio; ICD, implantable cardioverter-defibrillator; ILR, implantable loop recorder; LGE, late gadolinium enhancement; LV, left ventricular; LVEF, left ventricular ejection fraction; MAE, mean absolute error; ML, machine learning; MRI, magnetic resonance imaging; NPV, negative predictive value; PPV, positive predictive value; RCT, randomized controlled trial.

Study (First Author, Year) [Ref]	Clinical Domain	Input Data	Model Type	Cohort Size	Validation	Main Performance	External Validation	Key Limitation
Hannun, 2019 [4]	Rhythm classification	Single-lead ambulatory ECG	Deep neural network (supervised)	91,232 recordings	Internal test set (consensus reference)	12-class AUC 0.97; F1 0.837 vs. cardiologist 0.780	Limited	Point estimates without CIs; single data source
Attia, 2019 [5]	Occult AF during sinus rhythm	12-lead ECG	CNN (supervised)	649,931 ECGs/180,922 patients	Internal test set	AUC 0.87 (single ECG); 0.90 (1-month window)	Lower externally (AUC ≈ 0.62)	Single center; discrimination falls in external cohorts
Perez, 2019 [6]	AF screening via wearable	Photoplethysmographic pulse + ECG patch	Rule-based algorithm	419,297 participants	Prospective, single-arm	PPV 0.84 among returned patches	N/A	Only ~21% of notified returned a patch; no cost-effectiveness
Alhusseini, 2020 [7]	Ablation target mapping (rotors)	Intracardiac electrograms	CNN (supervised)	35 patients	Internal, expert-compared	95% accuracy; C-statistic 0.96	No	Very small cohort; proof-of-concept
Deisenhofer, 2025 (TAILORED-AF) [8]	AI-guided ablation of persistent AF	Intracardiac electrogram dispersion	Ensemble (gradient boosting + CNN)	370 randomized	Multi-center RCT (26 sites)	Single-procedure freedom from AF 88% vs. 70% (HR 0.30)	Multi-center	No difference in any-atrial-arrhythmia after one procedure; ~2× procedure time; model frozen 2020
Monteiro, 2026 [9]	AF recurrence after ablation	Clinical/imaging/electrophysiological	ML (various)	17-study meta-analysis	Meta-analysis	Mean AUC ≈ 0.81	Few	Substantial heterogeneity; mostly internal testing
Rosier, 2021 [10]	ILR alert triage	ILR-detected episodes	ML reclassification	370 patients/3755 episodes	Multi-center evaluation	43% of analyzable episodes reclassified as normal	No	Workload metric, not clinical outcomes
Mittal, 2021 [11]	ILR AF filtering	ILR-detected AF episodes	Deep-neural-network filter	1500 episodes	Validation study	PPV 53.9% → 74.5%	No	Adjudication endpoint only
Boehmer, 2017 (MultiSENSE) [12]	HF decompensation (CIED)	Multi-sensor device data	Composite index (HeartLogic)	900 patients	Prospective validation	70% of HF events detected; 34-day median warning	No	Alert-based; no randomized trial of the intervention
Sammut-Powell/Ahmed, 2022 (Triage-HF) [13]	HF hospitalization risk (CIED)	Up to nine device-derived parameters	Bayesian belief network	429 patients	Real-world validation	High status: sensitivity 37.3%, specificity 86.2%; NPV 97.5%	Yes (multi-cohort)	High false-positive alert rate
Kalscheur, 2018 (COMPANION) [14]	CRT outcome prediction	Pre-implant clinical variables	ML	595 CRT-defibrillator	Trial-derived model	AUC 0.74	No	Modest discrimination
Tokodi, 2020 (SEMMELWEIS-CRT) [15]	CRT mortality prediction	33 clinical variables	ML	1510 patients	Internal; later external	C-statistic ≈ 0.69	Yes (subsequent study)	Single-center derivation
Cikes, 2019 [16]	CRT responder phenogrouping	Clinical + echocardiographic deformation	Unsupervised (kernel learning + k-means)	1106 (MADIT-CRT)	Trial cohort	Responder phenogroups HR 0.35/0.36	No	Proof-of-concept
Popescu, 2022 (SSCAR) [17]	Arrhythmic death/ICD selection	LGE cardiac MRI + clinical	DL + survival analysis	Multi-site (ischemic CM)	Internal + independent test	C-index 0.83/0.74	Yes (independent test)	Ischemic CM only; imaging-dependent
Giudicessi, 2021 [18]	Long QT syndrome/QTc	12-lead and mobile ECG	Deep neural network	1.6 M ECGs/538,200 patients	Internal + mobile device	QTc ≥500 ms AUC 0.97; mobile 80%/94%	Partial (mobile device)	Single health system
Lin, 2020 (ECG12Net) [19]	Dyskalemia (K+)	12-lead ECG	Deep neural network	66,321 records	Internal + human comparison	Hyperkalemia AUC 0.958; K+ MAE 0.531 mEq/L	No	Single center; external validation needed
Attia, 2019 (low LVEF) [20]	LV systolic dysfunction	12-lead ECG	CNN	52,870 (test set)	Internal test set	AUC 0.93; sensitivity 86.3%, specificity 85.7%	Subsequent prospective work	Single-center development

## Data Availability

No new data were created or analyzed in this study. Data sharing is not applicable to this article.
